# Endemicity, disability and neglect: Leprosy in Colombia 2007–2020

**DOI:** 10.1371/journal.pntd.0013514

**Published:** 2025-09-22

**Authors:** Carlos Arango-Úsuga, Jesús Ochoa, Doracelly Hincapié-Palacio, Alba León

**Affiliations:** Universidad de Antioquia - Facultad Nacional de Salud Pública, Medellín, Colombia; London School of Hygiene and Tropical Medicine, UNITED KINGDOM OF GREAT BRITAIN AND NORTHERN IRELAND

## Abstract

**Background:**

Disability due to leprosy in Colombia is a neglected public health problem. This work aims to describe the magnitude of leprosy in Colombia, the spatiotemporal distribution of the disability and explore the potential relationship between individual and treatment delay characteristics with degrees of disability.

**Methods:**

Official leprosy data in Colombia between 2007 and 2020 were analyzed. The distribution of the grade 2 disability (G2D) rate was estimated. A Poisson spatiotemporal model was constructed to form clusters of municipalities with risk of G2D. A multinomial logistic regression model was used to quantify the relationships between patient characteristics and disability grades 1 (G1D) and 2 (G2D).

**Results:**

During the fourteen-year period, 5240 leprosy cases were registered (median age: 51 years, IQR: 35–63), of which 63.8% (n = 3341) were men. The proportion of multibacillary forms was 65.9% (n = 3453), 47.1% (n = 2468) for grade 0 disability (G0D), 18.4% (n = 966) for G1D and 9.7% (n = 507) for G2D. Three clusters and 10 municipalities were detected for the municipal rate of G2D. The national rate of G2D ranged between 0.03/100,000 inhabitants in 2010 and 0.05/100,000 inhabitants in 2020. The prevalence ratio of G1D and G2D was significant in individuals aged 60 years or older, men, from the subsidized or uninsured health system, who had relapses, multibacillary type and in whom the delay between the onset of symptoms and treatment was 7–12 years.

**Conclusion:**

In the context of leprosy elimination in Colombia, the prevalence of disability is high and heterogeneous in time and space. It is recommended to coordinate the necessary actions to “revitalize” the active epidemiological surveillance in the prioritized municipalities and strengthen the program including improving early detection, treatment and individual follow-up of patients with disabilities.

## Author summary

Leprosy is a global public health challenge due to its risk of disability. Although Colombia has achieved elimination status and disease indicators have remained stable, leprosy remains a neglected disease. In this study, we described the characteristics of patients with leprosy and the spatiotemporal evolution of the most severe disability from Colombia. Our findings show that its presence in the country is persistent and that the degree of disability varies considerably by region. From the analysis, we identified concerning patterns in the rate of grade 2 disability (i.e., visible deformities), highlighting the need to improve access to early diagnosis and strengthen prevention strategies. In this research, we seek to provide key information to optimize epidemiological surveillance programs and reduce disease-related complications, contribute to knowledge about leprosy in Colombia, and provide evidence to refine prevention and treatment strategies, in order to mitigate the impact of the disease on affected communities.

## Introduction

Leprosy or Hansen’s disease is one of the twenty neglected tropical diseases (NTDs) defined by the World Health Organization (WHO) [1]. Leprosy has a long history in the Americas, long before the arrival of Europeans. Some authors report the presence of ancient strains of *M. lepromatosis* in North and South America during the late Holocene period [2]. Leprosy is a very complex chronic communicable disease particularly because of its biological and cultural dimensions. The clinical presentation is diverse, ranging from the presence of one or more skin lesions to neuropathy (overt or silent) that is associated with deformities and disabilities, causing suffering and exclusion, particularly in poor communities [3].

The WHO defines disability as “a broad term covering any impairment, activity limitation or participation restriction affecting a person” [4]. The WHO classifies disability in leprosy patients into three grades (0, 1, and 2) in eyes, hands, and feet. Grade 0 indicates no loss of sensibility or visible deformity; grade 1 indicates loss of sensibility without visible deformity; and grade 2 indicates visible deformity [5].

Worldwide, leprosy remains a public health problem. In 2021, 140,594 new cases were reported globally, corresponding to a detection rate of 17.83 per million inhabitants [6]. This represented a 10.2% increase from 2020, when 128,405 cases were recorded. Additionally, 8,492 new cases of grade 2 disability (G2D) were reported, with a G2D rate of 1.1 per million population—this rate reflects diagnoses of patients with visible deformities [6]. The COVID-19 pandemic affected leprosy programs in most countries, early diagnosis and ongoing surveillance were disrupted due to mobility and screening restrictions. Most health services were reduced to maintaining essential services and health workers were reassigned to other services [6]. The impact on national leprosy programs was evident, as global statistics indicate.

Leprosy remains both a collective and individual problem, often overlooked worldwide, and Colombia is no exception. Obregon D in his article on the rhetoric of hygiene and science, 1920–1940, pointed out that “the advance of scientific investigation of leprosy was not supported by an encompassing project of social reform to modify the conditions of poverty, which contributed to the expansion of the disease” [7]. Despite recent publications showing “stable” indicators of the disease in Colombia [6,8], disability is still a sign of neglect in some regions of the country.

In endemic areas, continued transmission within communities, even with reduced prevalence, can result in a steady stream of new cases, some with G2D. Leprosy-associated disability may persist despite disease elimination due to delayed care-seeking and delayed diagnosis and treatment. Leprosy-associated disability in newly diagnosed cases may be related to delayed care-seeking in weakened health systems. Poverty, lack of education, social stigma, and misconceptions about disease and medical care can all contribute to delayed care-seeking [9,10]. Thus, untreated leprosy cases due to delayed diagnosis sustain community transmission of the disease [11].

G2D at diagnosis is widely recognized as an indicator of delayed case detection and of a hidden leprosy burden [11–13]. Monitoring the rate of new cases with G2D per million population provides a measure not only of program performance in early detection but also of the morbidity attributable to leprosy [12]. Persistently high G2D rates have been associated with socioeconomic vulnerability, stigma, and barriers to timely access to health services [10,14]. Despite the publication of valuable annual official reports, it is necessary to systematize and deepen the reflection on the magnitude and distribution of leprosy disability to identify municipalities that need to strengthen their prevention and control programs. In Colombia, the maximum degree of disability of each patient reported is recorded, but the anatomical location (eyes, hands, or feet) where the hypoesthesia or deformity occurs is not specified [5].

Ogunsumi DO et al. propose a standardized approach to classifying leprosy endemicity in countries and regions to improve communication and planning of control programs [15]. This research adopts this approach for Colombia, applying the metrics and method to measure and classify leprosy disability at the subnational level. The aim of this work was to characterize the spatiotemporal pattern of the most severe disability (G2D) due to leprosy and to explore the potential relationship between individual and treatment delay characteristics with degrees of disability.

## Methods

### Ethics statement

This study was approved by the Bioethics Committee of the National School of Public Health of Universidad de Antioquia (session 283 of March 25, 2022 – Code: 21030002-0065-2022).

### Study design and data source

A cross-sectional analysis based on surveillance data was conducted from 2007 to 2020 in Colombia. The study population included all leprosy cases reported to the National Public Health Surveillance System (SIVIGILA, by its Spanish acronym), which served as the main source of information. As the entire dataset was analyzed, no sampling procedure was applied. Records with unknown department of residence, cases from abroad, and those with age less than 30 days or age equal to zero were excluded. The study followed the RECORD statement [9] (S1 Appendix), and data quality was assessed through internal consistency checks and a completeness review (S2 Appendix).

### Definitions

The diagnosis of leprosy in Colombia is basically clinical [16,17], and is confirmed by smear microscopy and biopsy in order to classify the patient and define treatment and follow-up. The identification of *Mycobacterium leprae* or *M. lepromatosis* by polymerase chain reaction (PCR) is available only in research laboratories [16,18].

Colombia follows the WHO operational definitions [17,19]. A case of leprosy presents at least one of the following characteristics:

(a)  Hypopigmented skin lesions with hypoesthesia.
(b)  Impaired peripheral nerves (clear hypoesthesia, or weakness in hands and feet or face, or functional disorders such as anhidrosis).
(c)  Presence of visible deformities.
(d)  Signs of the disease with observation of bacilli in a skin smear or histopathological confirmation and who also needs treatment for leprosy.

Patients may be classified [17,19] as:

(a)  Paucibacillary leprosy: leprosy case with 1–5 skin lesions without presence of bacilli on a skin smear (Bacillary index = 0).
(b)  Multibacillary leprosy: case of leprosy with more than 5 skin lesions or nerve involvement (pure neuritis or any number of skin lesions) or presence of bacilli in a skin smear irrespective of the number of skin lesions (Bacillary index > 0).

The physical disability due to leprosy is defined in three categories [20,21]: Grade 0: no anesthesia or deformity of the eyes, hands or feet. Grade 1: decreased or absent corneal sensitivity, anesthesia in hands or feet without visible deformity. Grade 2: presence of deformities or visible eye damage (lagophthalmos and/or ectropion, trichiasis, corneal opacity), ulcers and traumatic lesions in hands or feet or mobile claw, bone resorption, foot drop and/or joint contracture.

### Spatiotemporal and statistical analysis

In the analysis, the distribution of the G2D rate indicator was described through thematic maps, using the parameter categories established in the systematic review and Delphi survey conducted by Ogunsumi DO et al. [15] for G2D rates: low (< 0.5/100,000 inhabitants); medium: (0.5 to 1/100,000 inhabitants), and high (> 1/100,000 inhabitants). This analysis of G2D was performed by department and municipality of residence. The denominators of the G2D rates were the population projections 2005–2017 and 2018–2050 published by the National Administrative Department of Statistics of Colombia (DANE, by its Spanish acronym) [22].

A spatiotemporal model was constructed using the Kulldorff statistic (Poisson distribution) [23]. This technique enables early detection of outbreaks or unusual increases in cases over time and space through a prospective space-time permutation scan statistic that does not require population-at-risk data [24]. Municipalities were prioritized by locating the highest number of new cases with G2D in the most recent years between 2007 and 2020. Subsequently, the G2D rate was calculated to define the order of priority according to the highest rate identified.

Using a multinomial logistic regression model, crude (cPR) and adjusted (aPR) prevalence ratios were calculated to estimate the relationships between individual and treatment delay characteristics with disability grades 1 and 2 as dependent variables. G0D was considered as the reference category.

Data processing and analysis were performed in Power BI (Microsoft), Excel (Microsoft) and Stata version 17 (StataCorp, College Station, TX, USA). Temporal and spatial analysis of new G2D cases was conducted using SaTScan version 10.1 (M. Kulldorff, Harvard Medical School and Information Management Services Inc.) and ArcGIS Desktop version 10.8 (Esri Inc., Redlands, CA, USA).

## Results

**Fig 1:**
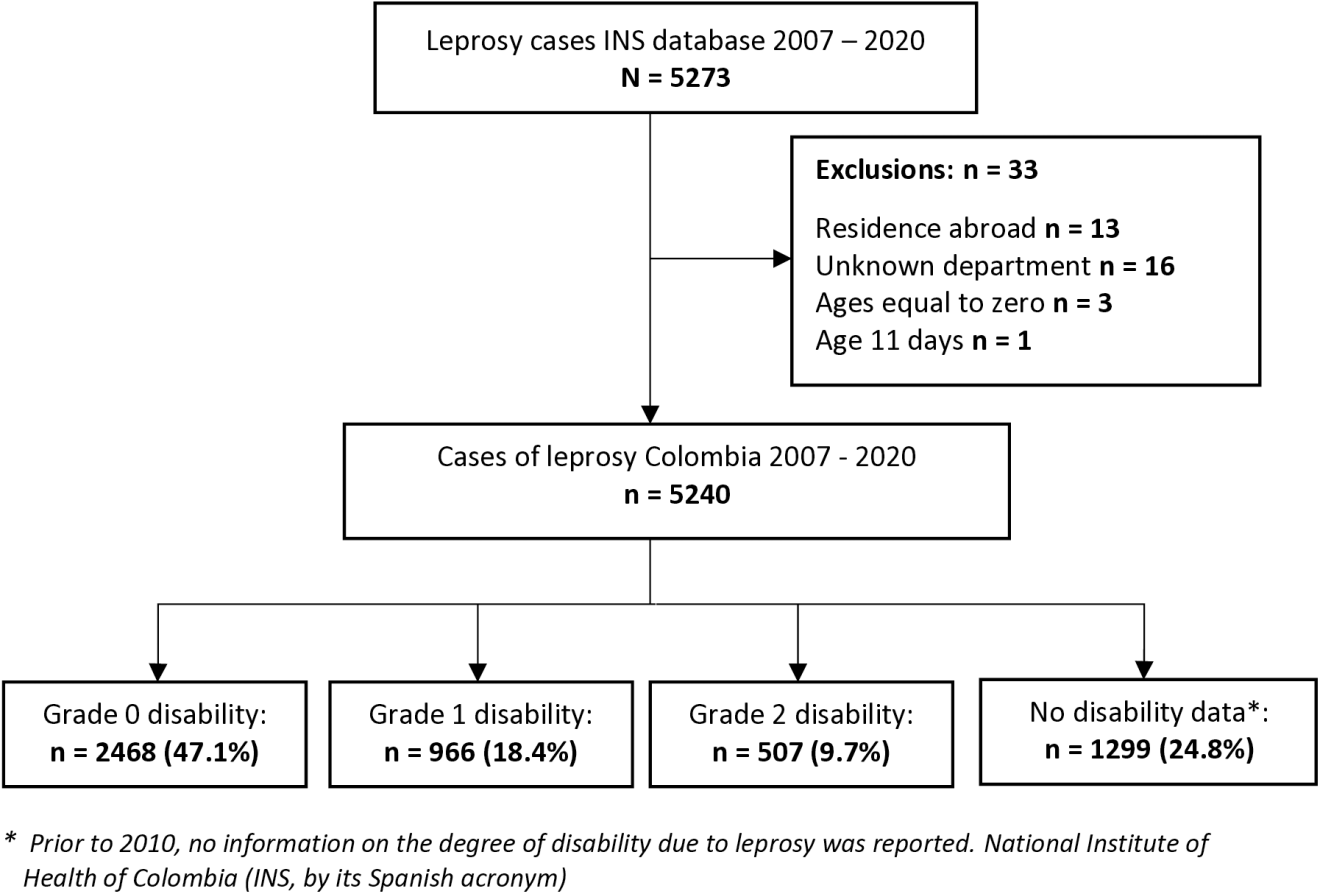
Flow chart of the study population, leprosy in Colombia 2007–2020.

Within the 14-year period, 5240 cases of leprosy were eligible for the study out of the 5273 reported to SIVIGILA. In the analysis of the quality of the data, 48/103 variables were found to be properly filled, 55 variables had one or more empty records or omitted data and of these only one variable had errors, the total number of symptomatic cohabitants was filled in erroneously on 2 occasions, it presented a format not in accordance with the format of the leprosy form (S2 Appendix). (Fig 1).

The cases were reported mainly in men 63.8% (n = 3341), aged 15–59 years, with a median age 51 years (IQR: 35–63), residents of the municipal capital, unqualified personnel, and uninsured or in the subsidized social security health care system. (Table 1).

**Table 1:** Demographic characteristics of leprosy cases by degree of disability Colombia, 2007– 2020^a^.

Characteristics	Total (n = 5240)		Grade 0 (n = 2468)		Grade 1 (n = 966)		Grade 2 (n = 507)	
	n	(%)	n	(%)	n	(%)	n	(%)
**Gender**								
Female	1899	(36.2)	979	(39.7)	325	(33.6)	127	(25.0)
Male	**3341**	**(63.8)**	**1489**	**(60.3)**	**641**	**(66.4)**	**380**	**(75.0)**
**Age in years**								
Median age (IQR)	**51 (35**–**63)**		**49 (34**–**61)**		**52 (38**–**64)**		**58 (43**–**70)**	
**Age group**								
< 15	133	(2.5)	79	(3.2)	16	(1.7)	8	(1.6)
15–59	**3436**	**(65.6)**	**1680**	**(68.1)**	**619**	**(64.1)**	**264**	**(52.1)**
≥ 60	1671	(31.9)	709	(28.7)	331	(34.3)	235	(46.4)
**Area of occurrence**								
Municipal headwaters	**3580**	**(68.3)**	**1743**	**(70.6)**	**633**	**(65.5)**	**326**	**(64.3)**
Rural	1042	(19.9)	431	(17.5)	211	(21.8)	115	(22.7)
Population center	618	(11.8)	294	(11.9)	122	(12.6)	66	(13.0)
**Occupation**								
Other occupations	916	(17.5)	488	(19.8)	155	(16.0)	60	(11.8)
Farmers and related	874	(16.7)	347	(14.1)	173	(17.9)	108	(21.3)
Elementary occupations, unskilled personnel	**3450**	**(65.8)**	**1633**	**(66.2)**	**638**	**(66.0)**	**339**	**(66.9)**
**Health system**								
Contributive/ exception/ special	1664	(31.8)	938	(38.0)	266	(27.5)	94	(18.5)
Subsidized/ Uninsured/ Indeterminate	**3576**	**(68.2)**	**1530**	**(62.0)**	**700**	**(72.5)**	**413**	**(81.5)**
**Ethnicity**								
Another	4595	(87.7)	2237	(90.6)	856	(88.6)	456	(89.9)
Black and Afro-Colombian populations	**556**	**(10.6)**	**187**	**(7.6)**	**97**	**(10.0)**	**43**	**(8.5)**
Indigenous	54	(1.0)	27	(1.1)	11	(1.1)	6	(1.2)
Raizal, palenquero^b^	35	(0.7)	17	(0.7)	2	(0.2)	2	(0.4)

^a^IQR, interquartile range. **Results highlighted in bold.**

^b^Raizal and Palenquero are recognized ethnic minorities with Afro-Caribbean roots in Colombia. Raizal communities are from San Andrés, Providencia, and Santa Catalina; Palenquero communities are descendants of African maroon settlements in San Basilio de Palenque.

From the point of view of case classification, 86.4% (n = 4527) were new cases with multibacillary predominance, 47.1% (n = 2468) were identified as G0D, 18.4% (n = 966) were G1D and 9.7% (n = 507) were G2D (Table 2).

**Table 2:** Clinical characteristics of leprosy cases by degree of disability, Colombia 2007 –2020^a^.

Characteristics	Total (n = 5240)		Grade 0 (n = 2468)		Grade 1 (n = 966)		Grade 2 (n = 507)	
	n	(%)	n	(%)	n	(%)	n	(%)
**Type of case**								
New	**4527**	**(86.4)**	**2233**	**(90.5)**	**823**	**(85.2)**	**420**	**(82.8)**
Relapse	525	(10.0)	184	(7.5)	110	(11.4)	73	(14.4)
Reinstatement for abandonment or recovered	98	(1.9)	51	(2.1)	33	(3.4)	14	(2.8)
**Epidemiological type**								
Paucibacillary	1689	(32.2)	886	(35.9)	272	(28.2)	89	(17.6)
Multibacillary	**3453**	**(65.9)**	**1577**	**(63.9)**	**694**	**(71.8)**	**416**	**(82.1)**
Data not available	98	(1.9)	5	(0.2)	0	(0.0)	2	(0.4)
**Clinical form**								
Undetermined	593	(11.3)	338	(13.7)	110	(11.4)	25	(4.9)
Tuberculoid	835	(15.9)	504	(20.4)	141	(14.6)	34	(6.7)
Diforma	369	(7.0)	198	(8.0)	68	(7.0)	16	(3.2)
Lepromatous	923	(17.6)	518	(21.0)	175	(18.1)	81	(16.0)
Neural	33	(0.6)	10	(0.4)	8	(0.8)	9	(1.8)
Other diagnoses	45	(0.9)	34	(1.4)	6	(0.6)	5	(1.0)
Data not available	2442	(46.6)	866	(35.1)	458	(47.4)	337	(66.5)
**Leprosy reaction**								
Type one	640	(12.2)	359	(14.6)	211	(21.8)	70	(13.8)
Type two	408	(7.8)	154	(6.2)	122	(12.6)	132	(26.0)
None	**2893**	**(55.2)**	**1955**	**(79.2)**	**633**	**(65.5)**	**305**	**(60.2)**
Data not available	1299	(24.8)	0	(0.0)	0	(0.0)	0	(0.0)

^a^IQR, interquartile range. **Results highlighted in bold.**

**Fig 2:**
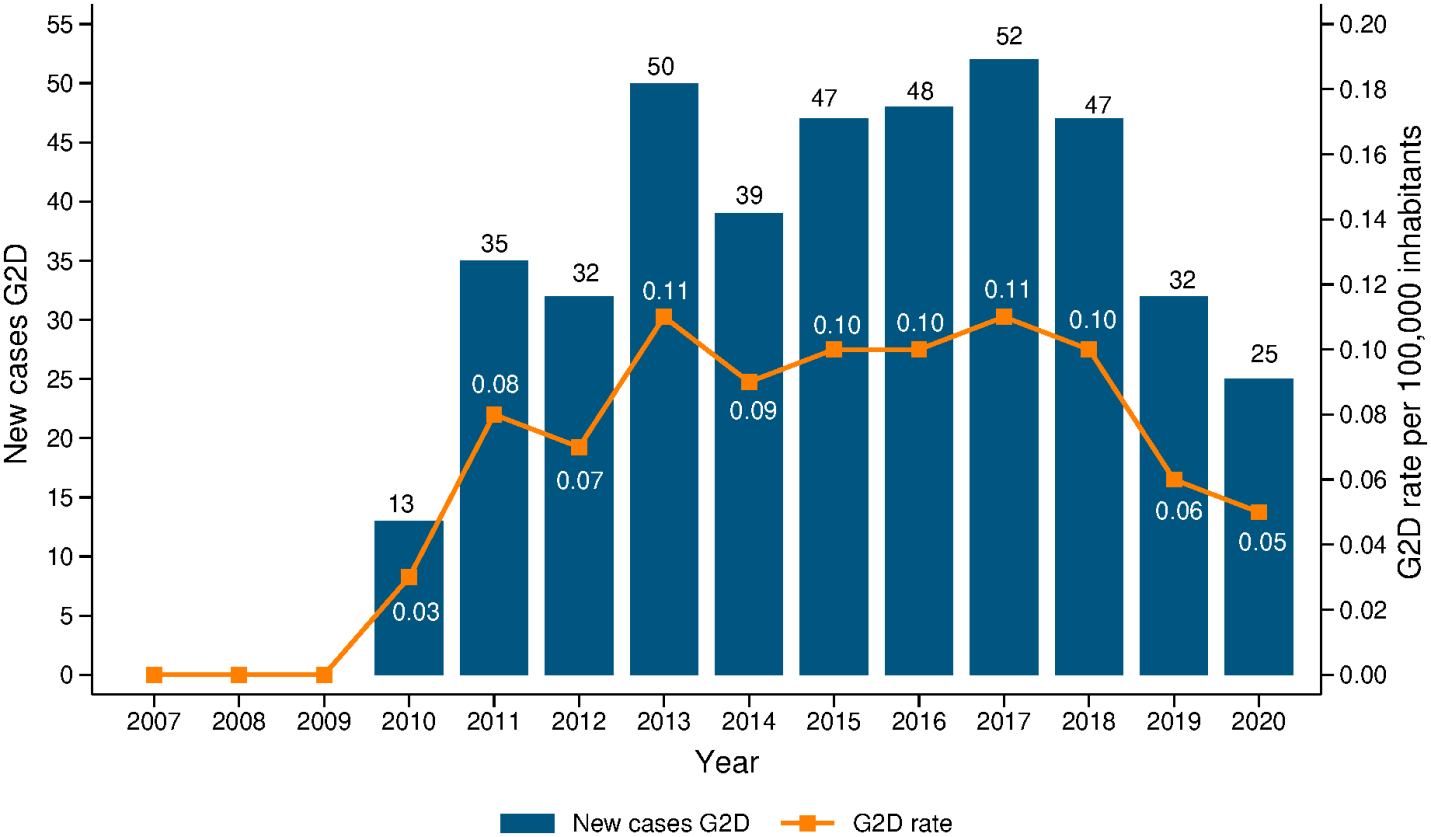
Annual national trend of grade 2 disability (G2D) among new cases and rates of G2D per 100 thousand inhabitants. **Colombia 2007**–**2020.**

**Fig 3:**
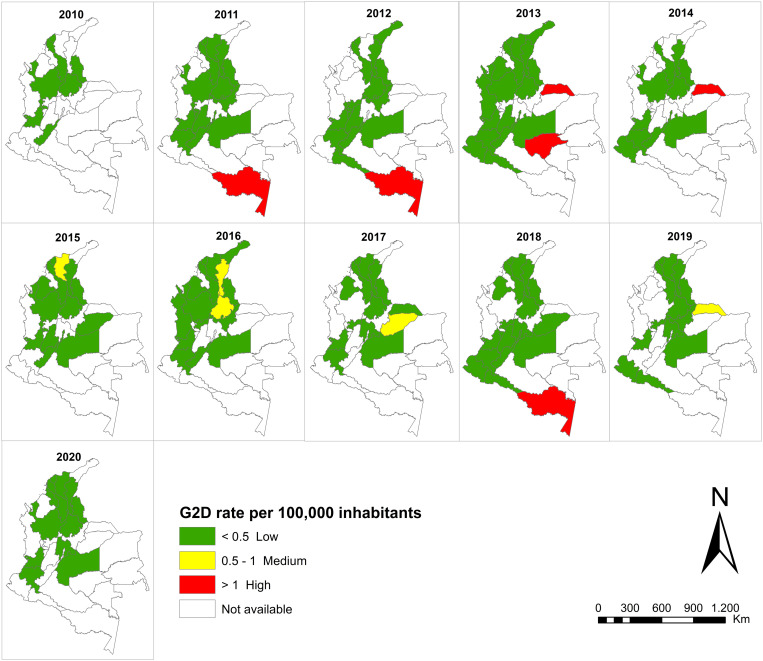
Geographic distribution of grade 2 disability rates (G2D) among new cases by department, Colombia in 2010–2020. Base layer maps of the country’s departments were obtained from open-access shapefiles provided by the National Administrative Department of Statistics of Colombia (DANE, by its Spanish acronym) through its geoportal: https://geoportal.dane.gov.co, under an open data policy. The authors confirm that the base maps can be published under the terms of the Creative Commons Attribution 4.0 International (CC BY 4.0) license: https://geoportal.dane.gov.co/acerca-del-geoportal/licencia-y-condiciones-de-uso/#gsc.tab=0. Maps were created using ArcGIS 10.8 (Esri Inc., Redlands, CA, USA).

The national rate of G2D ranged between 0.03/100,000 inhabitants in 2010 and 0.05/100,000 inhabitants in 2020 (Fig 2). High rates of G2D were observed in the departments of Amazonas (years 2011, 2012 and 2018), Guaviare (2013), and Arauca (2013 and 2014). With medium ranking, the rates of G2D of the departments of Magdalena (2015), Cesar and Santander (2016), Casanare (2017), and Arauca (2019) (Fig 3).

**Fig 4:**
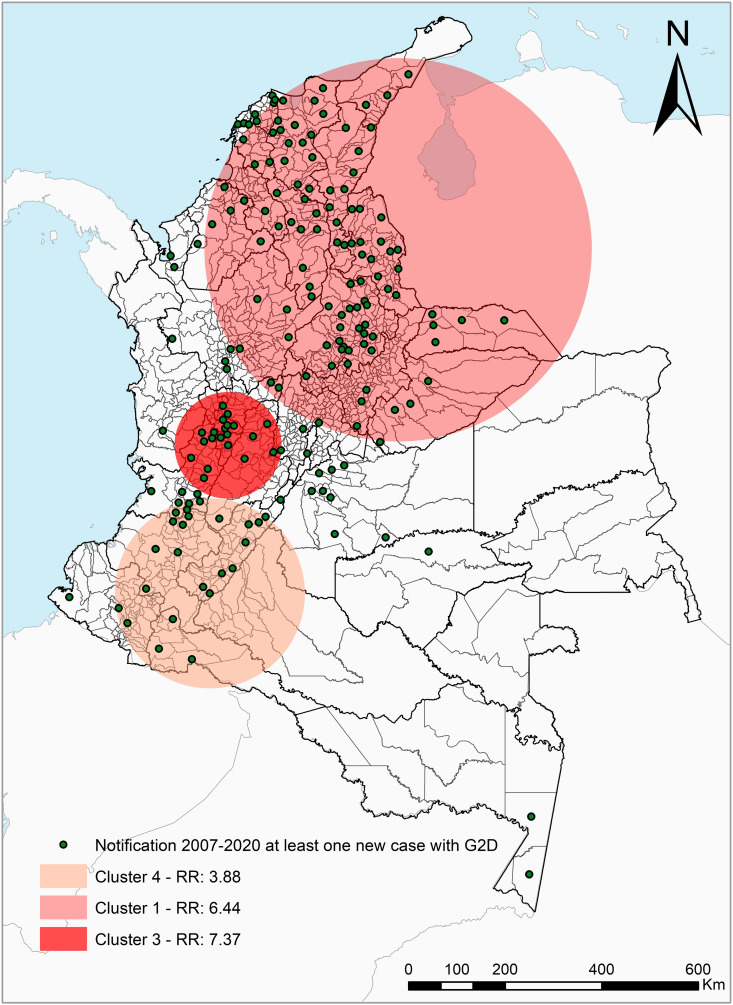
Spatial-temporal distribution of clusters of new cases with G2D, Colombia 2010–2020. Department and municipality boundaries were obtained from open-access shapefiles provided by the National Administrative Department of Statistics of Colombia (DANE, by its Spanish acronym) through its geoportal: https://geoportal.dane.gov.co, and are available under a Creative Commons Attribution 4.0 International (CC BY 4.0) license: https://geoportal.dane.gov.co/acerca-del-geoportal/licencia-y-condiciones-de-uso/#gsc.tab=0. The base map was created using physical and cultural vector data from Natural Earth (https://www.naturalearthdata.com), which are in the public domain and may be used without restriction: https://www.naturalearthdata.com/about/terms-of-use/. Maps were created using ArcGIS 10.8 (Esri Inc., Redlands, CA, USA).

For the G2D rate, three clusters were detected, corresponding to clusters 1, 3 and 4. Cluster 1 (RR = 6.44, p < 0.05) included 104 municipalities, located in 15 departments in the northeast of the country. Cluster 3 (RR = 7.37, p < 0.05) involved 21 municipalities from the departments of Caldas, Cundinamarca, Quindío, Risaralda, Tolima, and Valle. Cluster 4 (RR = 3.88, p < 0.05) included 24 municipalities from the departments of Cauca, Huila, Nariño, Putumayo, Tolima, and Valle (Fig 4, Table 3 and S3 Appendix).

**Table 3:** Spatio-temporal cluster of new cases with G2D due to leprosy in Colombia. 2007–2020.

Cluster	Number of municipalities	Observed cases	Cases expected	RR	Time period
**Cluster 1**	104	177	42.88	6.44	2013/1/1–2019/12/31
**Cluster 3**	21	24	3.43	7.37	2013/1/1–2019/12/31
**Cluster 4**	24	32	8.74	3.88	2012/1/1–2018/12/31

The municipality that presented the highest rate of G2D during the study period was the municipality of Contratación in Santander (in 2016 were 194.5 cases per 100 thousand inhabitants), other six municipalities were classified as high endemic (G2D rate > 1), and three municipalities were classified as medium endemic (G2D rates between 0.5 and 1). The prioritization also included standing out in the most recent years, the municipalities of Zambrano Bolivar (2019, 16.51 per 100 thousand inhabitants), Tuluá Valle (2019, 0.93 per 100 thousand inhabitants), and Aguachica Cesar (2020, 1.69 per 100 thousand inhabitants) (Table 4).

**Table 4:** Prioritized municipalities according to the highest rate of disability registered between 2007 and 2020^a^.

Cluster	Year	Municipality	Department	G2D Cases	G2D rate^b^
Cluster 1	2016	Contratación	Santander	7	194.50
Cluster 3	2017	Agua de Dios	Cundinamarca	3	26.49
Cluster 1	2019	Zambrano	Bolívar	2	16.51
Cluster 1	2012	Abrego	Norte de Santander	2	6.63
Cluster 1	2018	Magangué	Bolívar	5	3.75
Cluster 1	2020	Aguachica	Cesar	2	1.69
Cluster 1	2019	Ocaña	Norte de Santander	2	1.61
Cluster 3	2019	Tuluá	Valle del Cauca	2	0.93
Cluster 1	2013	Valledupar	Cesar	3	0.68
Cluster 1	2017	Bucaramanga	Santander	3	0.53

^a^Municipalities prioritized by the highest number of new cases with G2D in the most recent year between 2007 and 2020.

^b^Rates of grade 2 disability (G2D) among new cases x 100 thousand inhabitants.

The multinomial logistic regression model identified as risk markers for leprosy disability being 60 years or older [G1D aPR: 2.1 (95%CI: 1.0–4.5) and G2D aPR: 2.5 (95%CI 1.0–6.7)], being male [G1D aPR: 1.2 (95% CI: 1.0–1.6) and G2D aPR: 1.5 (95%CI: 1.1–2.1)] after adjusting for age group, health regimen, case type, epidemiological classification, and time from symptom onset to treatment (Table 5). Other risk factors identified were not having social security in health [G1D aPR: 1.7 (CI95%: 1.4–2.2), G2D aPR: 2.0 (CI 95%: 1.4–2.7)]; having a relapse [G1D aPR: 1.6 (CI 95%: 1.1–2.3), G2D aPR: 2.4 (CI 95%: 1.5–3.6)]; being classified as multibacillary [G1D aPR: 1.3 (CI 95%: 1.0–1.7), G2D aPR: 1.8(CI95%: 1.3–2.6)] and having time lag between onset of symptoms and treatment of 7–12 years [G1D aPR: 2.0 (CI95%: 1.1–3.5), G2D aPR: 5.7 (CI 95% 3.2–9.8)] (Table 5).

**Table 5:** Risk markers related to disability in patients with leprosy in Colombia 2007–2020^a^.

Characteristics	Bivariate analysis				Multivariate analysis			
	Grade 1^b^		Grade 2^b^		Grade 1^b^		Grade 2^b^	
	PR	(95% CI)	PR	(95% CI)	aPR	(95% CI)	aPR	(95% CI)
***Individual characteristics***								
**Gender**								
Female	Ref.		Ref.		Ref.		Ref.	
Male	**1.3**	**(1.1–1.5)**	**2.0**	**(1.6–2.4)**	**1.2**	**(1.0–1.6)**	**1.5**	**(1.1–2.1)**
**Age group**								
< 15	Ref.		Ref.		Ref.		Ref.	
15–59	1.8	(1.1–3.1)	1.6	(0.7–3.2)	1.8	(0.8–3.7)	1.2	(0.5–3.2)
≥ 60	**2.3**	**(1.3–4.0)**	**3.3**	**(1.6–6.9)**	**2.1**	**(1.0–4.5)**	**2.5**	**(1.0–6.7)**
**Area of occurrence** ^c^								
Municipal headwaters	Ref.		Ref.					
Rural	1.3	(1.1–1.6)	1.4	(1.1–1.8)				
Population center	1.1	(0.9–1.4)	1.2	(0.9–1.6)				
**Occupation** ^c^								
Other occupations	Ref.		Ref.					
Farmers and related	1.6	(1.2–2.0)	2.5	(1.8–3.6)				
Elementary occupations, unskilled personnel	1.2	(1.0–1.5)	1.7	(1.3–2.3)				
**Health system**								
Contributive/ exception/ special	Ref.		Ref.		Ref.		Ref.	
Subsidized/ Uninsured/ Indeterminate	**1.6**	**(1.4–1.9)**	**2.7**	**(2.1–3.4)**	**1.7**	**(1.4–2.2)**	**2.0**	**(1.4–2.7)**
**Ethnicity** ^c^								
Another	Ref.		Ref.					
Black and Afro-Colombian populations	1.4	(1.0–1.8)	1.1	(0.7–1.6)				
Indigenous	1.1	(0.5–2.2)	1.1	(0.4–2.7)				
Raizal, Palenquero	0.3	(0.1–1.3)	0.6	(0.1–2.5)				
***Clinical characteristics***								
**Type of case**								
New	Ref.		Ref.		Ref.		Ref.	
Relapse	**1.6**	**(1.3–2.1)**	**2.1**	**(1.6–2.8)**	**1.6**	**(1.1–2.3)**	**2.4**	**(1.5–3.6)**
Reinstatement for abandonment or recovered	1.8	(1.1–2.7)	1.5	(0.8–2.7)	0.9	(0.5–1.7)	0.6	(0.3–1.5)
**Epidemiological type**								
Paucibacillary	Ref.		Ref.		Ref.		Ref.	
Multibacillary	**1.4**	**(1.2–1.7)**	**2.6**	**(2.1–3.3)**	**1.3**	**(1.0–1.7)**	**1.8**	**(1.3–2.6)**
**Years from symptom onset to treatment**								
< 1	Ref.		Ref.		Ref.		Ref.	
1–6	1.2	(0.9–1.5)	1.1	(0.9–1.5)	1.2	(1.0–1.6)	1.3	(0.9–1.7)
7–12	**2.0**	**(1.1–3.5)**	**5.1**	**(3.0–8.6)**	**2.0**	**(1.1–3.5)**	**5.7**	**(3.2–9.8)**

^a^PR: prevalence ratio, aPR: adjusted prevalence ratio, 95% CI: 95% confidence interval, Ref: reference category. **Statistically significant results highlighted in bold.**

^b^Multinomial logistic regression. The reference category is disability degree 0.

^c^Variable excluded from the final model.

## Discussion

This study analyzed the grade 2 disability in Colombia between 2007 and 2020, one of the main indicators of leprosy endemic in Colombia. This work outlined an analysis path in line with the proposal for leprosy elimination by 2030 (the WHO Global Leprosy Strategy 2021–2030: “Towards zero leprosy”), identifying the municipalities where departmental and municipal programs should (ideally) reach to guide patients and their families.

Leprosy remains a neglected disease in Colombian public health programs, despite a recorded prevalence of less than one case per 10,000 inhabitants at the national level since 1997, probably earlier [17,25]. Gussow Z, in his classic text “Leprosy, racism and public health” described in 1989 the pattern of leprosy in endemic regions that included a higher frequency of the disease in men, predominance of multibacillary (MB) forms, location of clusters of cases in the municipalities and presence of childhood leprosy [26].

This endemic pattern coincides with the data presented here in these 14 years of leprosy in Colombia. The so-called “national stability” of the endemic and the pattern described in Colombia also coincide with a study on the epidemiological behavior of leprosy conducted between 2011–2020 in several Latin American countries, showing the highest occurrence of cases in men, MB forms and G2D was more frequent in Brazil [27]. A spatiotemporal study conducted in Brazil of all new cases of leprosy with G2D at diagnosis between 2001 and 2022, detected a similar behavior to that described (97.4% aged > 14 years, 70.2% male, 87.6% with low educational level and 90.2% multibacillary) [28].

This pattern of predominance of MB forms has implications for the follow-up of patients, their contacts, and epidemiological surveillance (i.e., the classification of patients and integrity of the information system). The specialized international literature indicates that reactional phenomena in leprosy are associated with neural damage and potential disability in more than 60% of patients with MB leprosy during the first 6 months of treatment [29]. The Colombian leprosy protocol considered as the basic orientation for rural physicians, lacks guidelines for the preventive and therapeutic management of reactional phenomena. The data presented in this study warns about this aspect due to the national frequency of multibacillary forms (65.9%) and the statistical relationship found between the degrees of disability (outcome) and this classification of the disease (exposure).

The appearance of disabilities reiterates that there are some gaps in the control of the disease. Indeed, the national predominance of multibacillary forms may indicate the occurrence of active transmission of the disease, as stated by Santana EMF [30]. Situations like this reflect contexts where transmission is intensified by the low detection of new cases and the delayed diagnosis and treatment, which increases disability, as shown by the national, departmental, and municipal leprosy rates.

These findings are consistent with a systematic review conducted between 2000 and 2021 from original studies of Asia (China, India, Nepal, Bangladesh, Myanmar), South America (Brazil, Colombia, Peru, Paraguay) and the United Kingdom, Authors identified healthcare factors related to delays in case detection in leprosy, including misdiagnosis as a central factor in delayed care, high number of pre-diagnosis consultations, lack of referral centers, healthcare personnel, and case detection methods. Geographic, financial, and organizational barriers were also identified. The authors recommended ensuring the sustainability of national leprosy control programs, including staff training within integrated health services [10].

In this sense, Jacobson RR stated that bacteriological healing cannot reverse established neural damage [31]. He provided guidance on the need for specialized medical care of reactional phenomena in an environment where experience and knowledge about leprosy are reduced. In 2013, Sales AM et al. in a study conducted in Brazil, concluded that patients with neural damage had a greater than 65% risk of worsening disability and that prompt treatment of reactional episodes allows prevention of disabilities [32].

Like other low-endemic countries, Colombia presents patterns of leprosy occurrence clustered at the subnational level (departmental or municipal). The spatial-temporal analysis of the indicator identified 149 municipalities and 3 clusters of G2D, particularly in the northeastern part of the country, as the area that has suffered the most from the disease [33], this departmental distribution has interesting historical particularities. It coincides, with some understandable variations due to the passage of time (and it is possible to see it in the maps), with what was published by Montoya and Flórez JB in 1906 and reproduced by Cardona H in 2011 [34], by Camargo D and Orozco LC in 1996 [35], by Cardona-Castro N in 2018 [36], and by the research presented here.

This study has some limitations. Systematizing and interpreting information on this disease in Colombia is difficult due to the heterogeneity in the quality of epidemiological surveillance information across departments and municipalities. These limitations include underreporting of cases, variations in diagnostic capacity across regions, and incomplete clinical records that may alter the accuracy of estimates of disease frequency and disability due to leprosy. Furthermore, it must be recognized that the model used may not be sensitive to detecting irregularly shaped clusters, in addition to its computational complexity [37]. However, this effort is valuable within the public health context for the discussion and methodological orientation of current proposals for leprosy elimination in Colombia.

## Conclusions

Disability due to leprosy in Colombia is heterogeneous, with annual variations in its departments and municipalities. The prevention of leprosy-related disabilities is a historical national debt. The lack of information on the disease is one of the symptoms of its invisibility and a neglect of historical nuances on which a good program and a clear epidemiological surveillance strategy can be reconstructed.

National indicators hide departmental and particularly municipal variations. It is recommended that the necessary actions be taken to promote the strategy of active epidemiological surveillance in the prioritized municipalities (particularly in contacts of MB forms) to define the individual follow-up of patients with G1D–G2D and the epidemiological field study of new cases. Additionally, it is important to distinguish disability in eyes, hands, and feet to guide the necessary measures to prevent the progression of disability, including early treatment and referral to specialized centers when necessary.

## Supporting information

S1 Appendix RECORD checklist. (DOCX)

S2 Appendix Data quality analysis. (DOCX)

S3 Appendix G2D cases in the departments and municipalities included in the clusters. (XLSX)
