# Supplementary material for: Endemicity, disability and neglect: Leprosy in Colombia 2007–2020
Source: PLoS Negl Trop Dis. 2025 Sep 22;19(9):e0013514. doi: 10.1371/journal.pntd.0013514 (PMC12453248; doi:10.1371/journal.pntd.0013514)
Supplement: S2 Appendix — (DOCX) [file pntd.0013514.s002.docx]

**Endemicity, disability and neglect: Leprosy in Colombia 2007 – 2020.**

**S2 Appendix. Data quality analysis.**

**Table.** Distribution of the data quality indicators evaluated for each of the available variables.

| Name | Variable | Quality indicators n (%) | | | |
| --- | --- | --- | --- | --- | --- |
|  |  | Valid data | | bad data | Not available |
| COD_EVE | event code | 5273 (100) | 0 (0) | | 0 (0) |
| FEC_NOT | date of notification | 5273 (100) | 0 (0) | | 0 (0) |
| SEMANA | week | 5273 (100) | 0 (0) | | 0 (0) |
| ANO | year | 5273 (100) | 0 (0) | | 0 (0) |
| COD_PRE | health care provider code | 5273 (100) | 0 (0) | | 0 (0) |
| COD_SUB | health care provider sub-index code | 5273 (100) | 0 (0) | | 0 (0) |
| TIP_IDE | type of identification | 5273 (100) | 0 (0) | | 0 (0) |
| EDAD | age | 5273 (100) | 0 (0) | | 0 (0) |
| UNI_MED | unit of measure of age | 5273 (100) | 0 (0) | | 0 (0) |
| SEXO | gender | 5273 (100) | 0 (0) | | 0 (0) |
| COD_PAIS_O | country code of occurrence | 5273 (100) | 0 (0) | | 0 (0) |
| COD_DPTO_O | department code of occurrence | 5273 (100) | 0 (0) | | 0 (0) |
| COD_MUN_O | municipality code of occurrence | 5273 (100) | 0 (0) | | 0 (0) |
| AREA | area of occurrence | 5273 (100) | 0 (0) | | 0 (0) |
| LOCALIDAD | locality | 1923 (36) | 0 (0) | | 3350 (64) |
| CEN_POBLA | town center | 487 (9) | 0 (0) | | 4786 (91) |
| VEREDA | village | 634 (12) | 0 (0) | | 4639 (88) |
| BAR_VER | neighborhood or sidewalk | 3017 (56) | 0 (0) | | 2256 (43) |
| OCUPACION | occupation | 5273 (100) | 0 (0) | | 0 (0) |
| TIP_SS | type of health plan | 5273 (100) | 0 (0) | | 0 (0) |
| COD_ASE | administrator code | 4639 (88) | 0 (0) | | 634 (12) |
| PER_ETN | ethnic affiliation | 5273 (100) | 0 (0) | | 0 (0) |
| GRU_POB | population group | 2254 (43) | 0 (0) | | 3019 (56) |
| GP_DISCAPA | population group - disabled | 5273 (100) | 0 (0) | | 0 (0) |
| GP_DESPLAZ | population group - displaced persons | 5273 (100) | 0 (0) | | 0 (0) |
| GP_MIGRANT | population group - migrants | 5273 (100) | 0 (0) | | 0 (0) |
| GP_CARCELA | population group - prison inmates | 5273 (100) | 0 (0) | | 0 (0) |
| GP_GESTAN | population group - pregnant women | 5273 (100) | 0 (0) | | 0 (0) |
| GP_INDIGEN | population group - indigent | 5273 (100) | 0 (0) | | 0 (0) |
| GP_POBICFB | population group - child population in ICBF | 5273 (100) | 0 (0) | | 0 (0) |
| GP_MAD_COM | population group - community mothers | 5273 (100) | 0 (0) | | 0 (0) |
| GP_DESMOVI | population group - demobilized persons | 5273 (100) | 0 (0) | | 0 (0) |
| GP_PSIQUIA | population group - population in psychiatric centers | 5273 (100) | 0 (0) | | 0 (0) |
| GP_VIC_VIO | population group - victims of armed violence | 5273 (100) | 0 (0) | | 0 (0) |
| GP_OTROS | population group - others | 5273 (100) | 0 (0) | | 0 (0) |
| COD_DPTO_R | department of residence code | 5273 (100) | 0 (0) | | 0 (0) |
| COD_MUN_R | municipality of residence code | 5273 (100) | 0 (0) | | 0 (0) |
| COD_DPTO_N |  | 5273 (100) | 0 (0) | | 0 (0) |
| COD_MUN_N |  | 5273 (100) | 0 (0) | | 0 (0) |
| FEC_CON | date of consultation | 5268 (99.91) | 0 (0) | | 5 (0.09) |
| INI_SIN | date of onset of symptoms | 5267 (99.89) | 0 (0) | | 6 (0.11) |
| TIP_CAS | initial case classification | 5273 (100) | 0 (0) | | 0 (0) |
| PAC_HOS | hospitalized | 5273 (100) | 0 (0) | | 0 (0) |
| FEC_HOS | date of hospitalization | 310 (6) | 0 (0) | | 4963 (94) |
| CON_FIN | final condition | 5273 (100) | 0 (0) | | 0 (0) |
| FEC_DEF | date of death | 22 (0.42) | 0 (0) | | 5251 (99.58) |
| AJUSTE | follow-up and final case classification (adjustment) | 5273 (100) | 0 (0) | | 0 (0) |
| FECHA_NTO | date of birth | 5210 (99) | 0 (0) | | 63 (1) |
| CER_DEF | death certificate number | 22 (0.42) | 0 (0) | | 5251 (99.58) |
| CBMTE | basic cause of death | 22 (0.42) | 0 (0) | | 5251 (99.58) |
| FEC_ARC_XL | date of creation of the flat file | 5273 (100) | 0 (0) | | 0 (0) |
| NOM_DIL_F | name of person filling out the record | 0 (0) | 0 (0) | | 5273 (100) |
| TEL_DIL_F | phone number of person making the record | 0 (0) | 0 (0) | | 5273 (100) |
| FEC_AJU | date of adjustment | 5273 (100) | 0 (0) | | 0 (0) |
| FM_FUERZA | military force | 10 (0.19) | 0 (0) | | 5263 (99.81) |
| FM_UNIDAD | military unit code | 10 (0.19) | 0 (0) | | 5263 (99.81) |
| FM_GRADO | military grade code | 10 (0.19) | 0 (0) | | 5263 (99.81) |
| VERSION | SIVIGILA version of the registry | 0 (0) | 0 (0) | | 5273 (100) |
| confirmados |  | 5273 (100) | 0 (0) | | 0 (0) |
| va_sispro |  | 5273 (100) | 0 (0) | | 0 (0) |
| Evento | name of the event | 5273 (100) | 0 (0) | | 0 (0) |
| Nombre_UPGD | name of the UPGD | 5272 (99.98) | 0 (0) | | 1 (0.02) |
| Departamento_ocurrencia | name of department of occurrence | 5273 (100) | 0 (0) | | 0 (0) |
| Municipio_ocurrencia | name of municipality of occurrence | 5273 (100) | 0 (0) | | 0 (0) |
| Departamento_residencia | name of department of residence | 5273 (100) | 0 (0) | | 0 (0) |
| Municipio_residencia | name municipality of residence | 5273 (100) | 0 (0) | | 0 (0) |
| Departamento_notificacion | name department of notification | 5273 (100) | 0 (0) | | 0 (0) |
| Municipio_Notificacion | name municipality of notification | 5273 (100) | 0 (0) | | 0 (0) |
| caso | type of case | 4728 (90) | 0 (0) | | 545 (10) |
| cla_lep | bacteriologic classification according to smear microscopy | 3776 (72) | 0 (0) | | 1497 (28) |
| clas_final | final classification according to histopathology result | 2498 (47) | 0 (0) | | 2775 (53) |
| baciloscop | smear microscopy | 3969 (75) | 0 (0) | | 1304 (25) |
| fec_bacilo | date of smear microscopy | 1933 (37) | 0 (0) | | 3340 (63) |
| res_bacilo | smear index result | 2878 (55) | 0 (0) | | 2395 (45) |
| fec_res_ba | date of smear microscopy result | 1855 (35) | 0 (0) | | 3418 (65) |
| biopsia | biopsy | 3969 (75) | 0 (0) | | 1304 (25) |
| fec_tom_bi | date biopsy taken | 1739 (33) | 0 (0) | | 3534 (67) |
| res_bio | histopathology result | 2972 (56) | 0 (0) | | 2301 (44) |
| fec_res_bi | date of biopsy result | 1739 (33) | 0 (0) | | 3534 (67) |
| max_gra_di | maximum degree of disability | 3968 (75) | 0 (0) | | 1305 (25) |
| pre_rea_le | leprosy reaction present | 3968 (75) | 0 (0) | | 1305 (25) |
| fec_ini_tr | date treatment started | 2766 (52) | 0 (0) | | 2507 (48) |
| met_hal | method of uptake | 2163 (41) | 0 (0) | | 3110 (59) |
| fue_con | possible source of infection | 2163 (41) | 0 (0) | | 3110 (59) |
| inv_campo | field investigation | 1405 (27) | 0 (0) | | 3868 (73) |
| fec_inv | date of investigation | 1155 (22) | 0 (0) | | 4118 (78) |
| tie_con | number of cohabitants | 2496 (47) | 0 (0) | | 2777 (53) |
| tot_con | total number of cohabitants | 11568 (30) | 0 (0) | | 3705 (70) |
| con_exa | total number of cohabitants examined | 1431 (27) | 0 (0) | | 3842 (73) |
| tot_con_si | total number of symptomatic cohabitants | 881 (16.71) | 2 (0.04) | | 4390 (83.25) |
| vac_bcg | total number of cohabitants vaccinated with BCG | 2087 (40) | 0 (0) | | 3186 (60) |
| observacio | observations and follow-up of the case | 446 (8) | 0 (0) | | 4827 (92) |
| NUM_IDE | identification number | 445 (9) | 0 (0) | | 4818 (91) |
| cla_clinic | clinical classification of the case | 2531 (48) | 0 (0) | | 2742 (52) |
| num_lesion | number of lesions | 2530 (48) | 0 (0) | | 2743 (52) |
| inic_trata | start of treatment | 1515 (28) | 0 (0) | | 3758 (71) |
| nacionalidad | nationality code | 614 (12) | 0 (0) | | 4659 (88) |
| nombre_nacionalidad | nationality name | 614 (12) | 0 (0) | | 4659 (88) |
| nom_grupo | name ethnic group | 614 (12) | 0 (0) | | 4659 (88) |
| estrato | socioeconomic stratum | 583 (11) | 0 (0) | | 4690 (89) |
| sem_ges | weeks of gestation | 614 (12) | 0 (0) | | 4659 (88) |
| fuente | source from which the information was received | 614 (12) | 0 (0) | | 4659 (88) |
| categoria_numero_lesiones | category number of injuries | 608 (12) | 0 (0) | | 4665 (88) |
